# Lethal Carmi syndrome (junctional epidermolysis bullosa-pyloric atresia) with a novel and recurrent compound integrin beta 4 heterozygous mutation: A case report

**DOI:** 10.1016/j.jdcr.2024.05.020

**Published:** 2024-05-27

**Authors:** Suci Widhiati, Nabila Kirtti Pradipta, Dewajani Purnomosari, Retno Danarti, Alifah Anggraini, Retno Palupi-Baroto, Niken Trisnowati

**Affiliations:** a Department of Dermatology and Venereology, Faculty of Medicine, Universitas Sebelas Maret/Dr Moewardi General Hospital, Surakarta, Indonesia; b Department of Dermatology and Venereology, Faculty of Medicine, Public Health, and Nursing, Universitas Gadjah Mada/Dr Sardjito General Hospital, Yogyakarta, Indonesia; c Department of Histology and Cell Biology, Faculty of Medicine, Public Health, and Nursing, Universitas Gadjah Mada, Yogyakarta, Indonesia; d Department of Child Health, Faculty of Medicine, Public Health, and Nursing, Universitas Gadjah Mada/Dr Sardjito General Hospital, Yogyakarta, Indonesia

**Keywords:** Carmi syndrome, epidermolysis bullosa, ITGB4, lethal, pyloric atresia

## Introduction

Carmi syndrome (CS) (OMIM:226730) is a rare severe disease characterized by junctional epidermolysis bullosa and pyloric atresia (JEB-PA), often with aplasia cutis and multisystem involvement, resulting in a poor prognosis.^1^ Epidermolysis bullosa with pyloric atresia arises from mutations in *ITGB4,* *ITGA6*, or *PLEC1* genes, which are essential for hemidesmosomes (HD) and anchoring filaments.^2^ CS is a specific case of JEB-PA, characterized by mutations in *ITGB4* and *ITGA6* in 78% of cases. However, other epidermolysis bullosa with pyloric atresia is caused by mutations in *PLEC1* genes which lead to epidermolysis bullosa simplex or collagen VII as epidermolysis bullosa dystrophic which is extremely rare.^3^ Over 100 mutations have been documented in these genes, leading to a spectrum of phenotypes.^1^ Here, we report a Javanese neonate with CS featuring aplasia cutis, renal abnormalities, and a novel nonsense pathogenic variant, p.Y1695X, causing a premature termination codon, with a variant of uncertain significance (VUS) of p.L1762P deemed unstable protein structure.

## Case report

A 19-day-old Javanese infant girl, born prematurely at 33 weeks with a low birth weight of 1860 grams due to complete placenta previa and antepartum bleeding, exhibited peculiar symptoms. She had extensive peeling skin, a left leg deformity, and vomited brown residue after milk intake. Tragically, her 2 older siblings, who also suffered from peeling skin since birth, passed away shortly after birth. Notably, her parents had no history of similar complaints or consanguinity.

**Fig 1:**
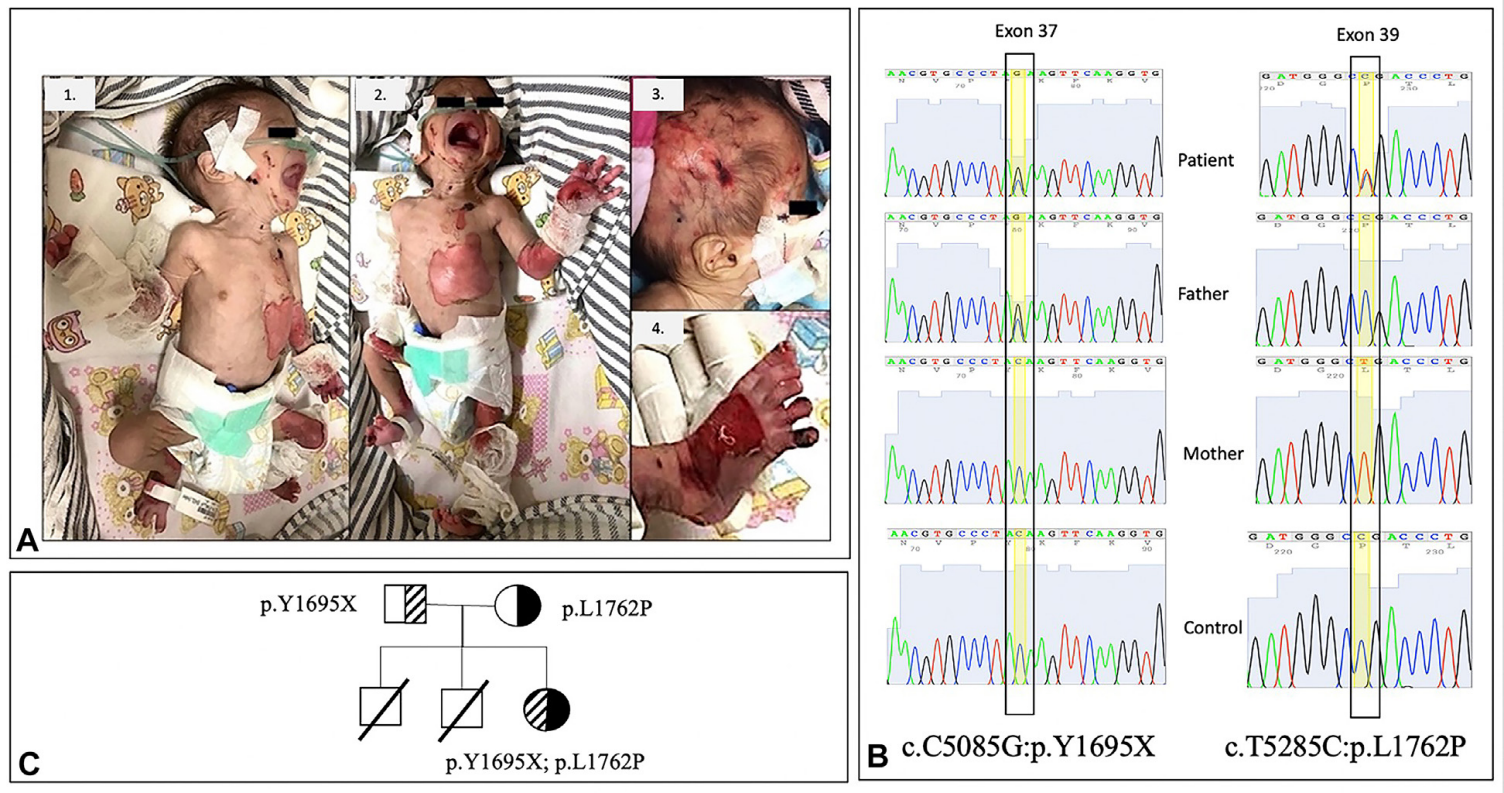
Clinical cutaneous characteristics of Carmi syndrome, skin detachment, and absence of skin extremity (**A**). A novel pathogenic variant of exon 37 in the patient and father, and also a “variant of uncertain significance” of exon 39 in the patient and mother (**B** and **C**).

**Fig 2:**
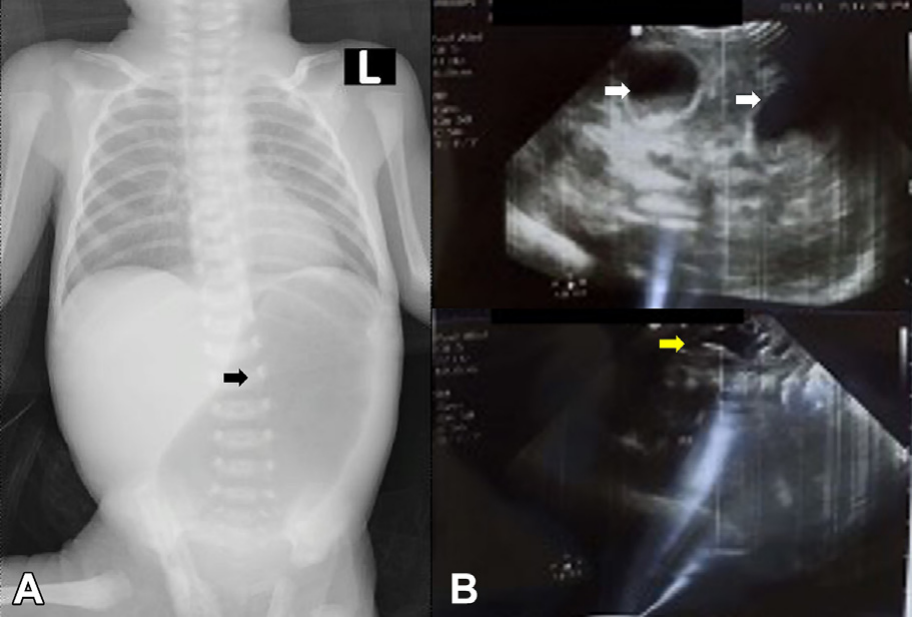
Radiological and ultrasonography examination. **A,** The X-ray confirmed a "single bubble appearance" suitable for pyloric atresia (*black arrow*). **B,** The ultrasonography examination identified bladder duplication (indicated by a *white arrow*) and left hydronephrosis characterized by the widening of the pelvicalyceal system and thinning of the calyx (indicated by the *yellow arrow*).

During the physical examination, numerous erosions of varying sizes were observed across her body, along with aplasia cutis on her left lower extremity, patches of alopecia, and oral mucosal erosions. There were no signs of bullae, finger adhesions, nail deformities, milia, excessive granulation tissue, scarring, palmoplantar keratoderma, poikiloderma, or ocular abnormalities (Fig 1). An abdominal X-ray revealed gastric dilatation (single bubble appearance), supporting the diagnosis of pyloric atresia. Furthermore, a renal ultrasound disclosed left hydronephrosis and bladder duplication (Fig 2). Subsequently, she underwent laparotomy and pyloroplasty surgeries by a pediatric surgeon. Unfortunately, the patient succumbed to septicemia 4 days after the surgical procedures.

Proband underwent whole exome sequencing, yielding pathogenicity predictions through SIFT, PolyPhen, Mutation Taster, LRT, Mutation Assessor, and FATHMM. Variants were classified using The American College of Medical Genetics and Genomics criteria via Varsome (http://varsome.com). Two *ITGB4* gene (NM_001005619) missense and nonsense variants were identified: c.T5285 C (p.L1762P) in exon 39 (rs871443) and c.C5085 G (p.Y1695X) in exon 37. c.T5285 C was classified as “variant of uncertain significance” (VUS) by The American College of Medical Genetics and Genomics. However, c.C5085 G was a novel pathogenic nonsense variant deemed “deleterious” by Mutation Taster and LRT. Notably, this novel variant was absent in Single Nucleotide Polymorphism Database, 1000Genomes Project, Genome-wide association study, and Exome Aggregation Consortium databases.

**Fig 3:**
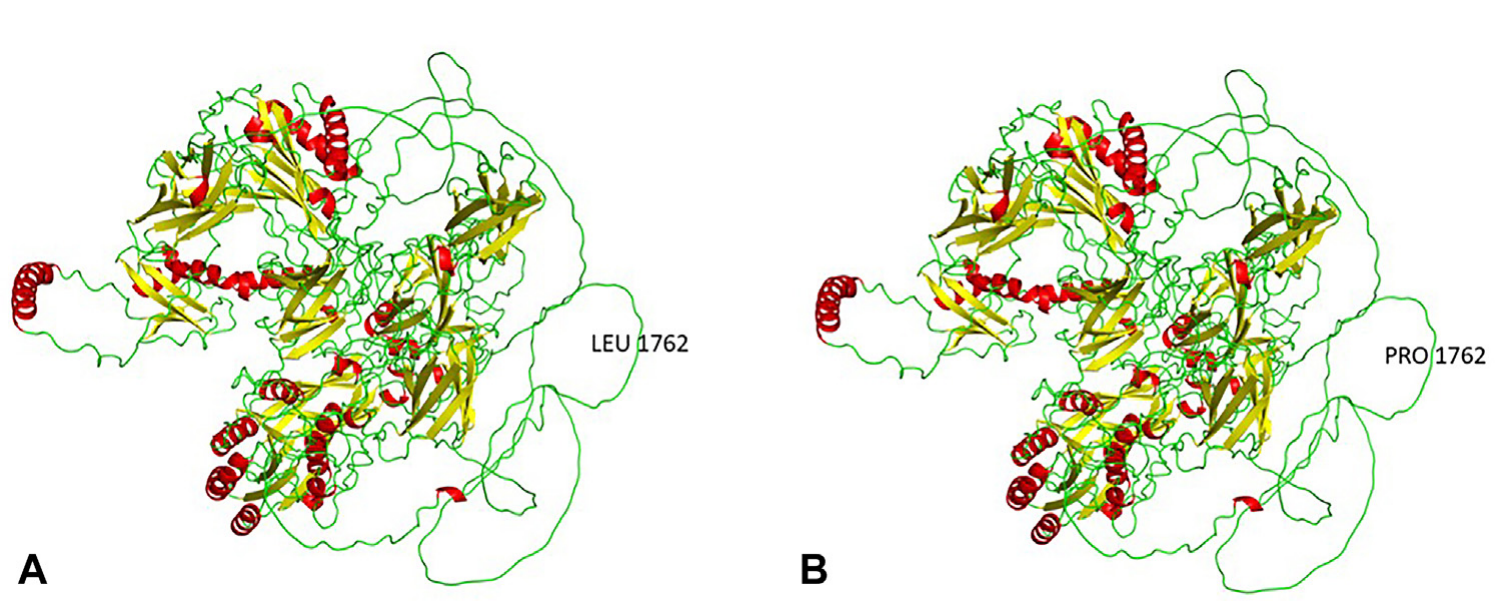
*ITGB4* protein structure. **A,** 1762Leu and (**B**) 1762Pro. *Red* (helix), *yellow* (sheet), *green* (loop). Changes at 1762 position still form a loop and do not alter the protein structure. However, the delta G score of 3.121 (delta G > 0) undergoes destabilizing effects.

Protein modelling of missense *ITGB4* p.L1762P was carried out using AlphaFold database (AlphaFold DB, https://alphafold.ebi.ac.uk), and was visualized using PyMol 2.5.7. The structure has a delta G score of 3.121 (delta score G > 0) measured with FoldX giving a destabilizing effects variant (Fig 3).^4^

Sanger sequencing was employed to validate these mutations, and ABI PRSM BigDye Terminator Cycle Sequencing Kit v3.1 was analyzed with the 4peaks application (http://nucleobytes.com/4peaks). The father carried p.Y1695X, the mother carried p.L1762P, and neither variant was present in the control. This inheritance pattern implicated an autosomal recessive transmission mode, with the patient inheriting p.L162P from her mother and p.Y1695X from her father.

## Discussion

We present a CS case accompanied by aplasia cutis and left hydronephrosis with bladder duplication, possibly arising from a heterozygous compound mutation, p.Y1695X and p.L1762P. CS, first coined by Carmi in 1968, is characterized by skin fragility, blistering, and aplasia cutis, along with pyloric atresia.^5^ Ureteral and renal anomalies, primarily hydronephrosis could also be seen as manifestations in epidermolysis bullosa with pyloric atresia.^3^ CS is frequently lethal in early infancy, as in our case. Additional features of CS, as reported by Mylonas et al in 2021, include aplasia cutis congenita (28%), urinary tract findings (21%), and ear and ocular findings (12% and 8%, respectively). CS cases have been identified worldwide, with reports from the United States, India, Saudi Arabia, Turkey, and Japan.^1^ However, no other case reports of JEB-PA or its eponym CS were found within the Indonesian Malay trait.

Cutaneous manifestations in CS involve peeling or blistering in localized or extensive areas, often appearing at birth or later. Additional features may include aplasia cutis, a fusion of skin between fingers and toes, nail dystrophy, and scarring alopecia.^5^ Aplasia cutis can be localized or extensive, and finger and toenail abnormalities with pseudo-syndactyly may occur. Joint deformations and contractures can limit mobility.^6^ In our case, multiple erosions covered almost the entire body, with extensive aplasia cutis on the left lower extremity, leading to contractures and limited mobility.

The *ITGB4* gene encodes an 1822 amino acid protein integral to HD. The proband carries a nonsense mutation in exon 37 (c.C5085 G (p.Y1695X)), resulting in reduced or absent integrin α6β4 expression.^7^ This pathogenic variant is located in the third fibronectin type III segment repeat and the carboxy-terminal half of the connecting segment, critical for binding between BP180 and integrin β4, vital for HD assembly. However, the single mutation in the father did not manifest clinically. The second variant, a VUS in exon 39 (c.T5285 C (p.L1762P)), had no clinical manifestation in the mother. However, the variant predicts to unstable protein structure of *ITGB4*. Pathogenic variants and VUS in *ITGB4* genes confirmed the diagnosis, predicting truncated and nonfunctional integrin β4 polypeptides. We hypothesize that the premature termination codon and pathogenic variant locations affecting integrin's functional domains contribute to JEB-PA's lethal phenotype. Premature termination codon mutations are often associated with poor prognosis.^2^

Integrin β4 is expressed in the gastrointestinal tract's epithelia, explaining congenital pyloric atresia due to α6β4 integrin absence.^8^ Mutations in integrin β4 subunits disrupting interactions with HD components can disassemble HD in the urinary tract.^9^ Another manifestation of heterozygous variants in *ITGB4* is autosomal dominant nail dystrophy, which is associated with von Willebrand factor type A domain (or beta I domain) the ligand binding site domain.^10^ Our patient exhibited JEB-PA with hydronephrosis and bladder duplication, in the third fibronectin type III domain consistent with literature reports. The manifestation of nail dystrophy could not be assessed due to aplasia cutis.

## Conclusion

The compound heterozygous *ITGB4* variant presented with a novel pathogenic variant, c.C5085 G (p.Y1695X), and a variant of uncertain significance, c.T5285 C (p.L1762P), resulting in the lethal characteristics of CS. This marks the first CS report within the Indonesian Malay population, and the novel mutation has to be documented in other databases.

## Declaration of generative AI and AI-assisted technologies in the writing process

While preparing this manuscript, the authors used chatGPT (chat.openai.com) to improve language and readability. After using this service, the authors reviewed and edited the content as needed and took full responsibility for the content of the publications.

## Conflicts of interest

None disclosed.

## References

[1] Mylonas K.S., Hayes M., Ko L.N., Griggs C.L., Kroshinsky D., Masiakos P.T. (2019). Clinical outcomes and molecular profile of patients with Carmi syndrome: a systematic review and evidence quality assessment. J Pediatr Surg.

[2] Ellis C., Eason C., Snyder A. (2021). Novel missense p.R252L mutation of ITGB4 compounded with known 3793+1G>A mutation associated with nonlethal epidermolysis bullosa-pyloric atresia with obstructive uropathy. JAAD Case Rep.

[3] Luo C., Yang L., Huang Z. (2023). Case report: a case of epidermolysis bullosa complicated with pyloric atresia and a literature review. Front Pediatr.

[4] Schymkowitz J., Borg J., Stricher F., Nys R., Rousseau F., Serrano L. (2005). The FoldX web server: an online force field. Nucleic Acids Res.

[5] Hicks T.D., Singh H., Mikhael M., Shah A.R. (2018). Carmi syndrome in a preterm neonate: a multidisciplinary approach and ethical challenge. Case Rep Pediatr.

[6] Matyas M., Miclea D., Zaharie G. (2021). Case report: uncommon association of ITGB4 and KRT10 gene mutation in a case of epidermolysis bullosa with pyloric atresia and aplasia cutis congenita. Front Genet.

[7] Birnbaum R.Y., Landau D., Elbedour K., Ofir R., Birk O.S., Carmi R. (2008). Deletion of the first pair of fibronectin type III repeats of the integrin beta-4 gene is associated with epidermolysis bullosa, pyloric atresia and aplasia cutis congenita in the original Carmi syndrome patients. Am J Med Genet A.

[8] Dang N., Klingberg S., Rubin A.I. (2008). Differential expression of pyloric atresia in junctional epidermolysis bullosa with ITGB4 mutations suggests that pyloric atresia is due to factors other than the mutations and not predictive of a poor outcome: three novel mutations and a review of the literature. Acta Derm Venereol.

[9] Liebert M., Washington R., Wedemeyer G., Carey T.E., Grossman H.B. (1994). Loss of co-localization of alpha 6 beta 4 integrin and collagen VII in bladder cancer. Am J Pathol.

[10] Malovitski K., Meijers O., Cohen-Barak E. (2022). Heterozygous variants in the integrin subunit beta 4 gene (ITGB4) cause autosomal dominant nail dystrophy. Br J Dermatol.

